# Prevalence of Violence Victimization and Perpetration Among Persons Aged 13–24 Years — Four Sub-Saharan African Countries, 2013–2015

**DOI:** 10.15585/mmwr.mm6815a3

**Published:** 2019-04-19

**Authors:** Elizabeth A. Swedo, Steven A. Sumner, Susan D. Hillis, George Aluzimbi, Rose Apondi, Victor O. Atuchukwu, Andrew F. Auld, Peter J. Chipimo, Martha Conkling, Okpewuru E. Egbe, McKnight S.H. Kalanda, Chabila C. Mapoma, Emma Phiri, Lydia N. Wasula, Greta M. Massetti

**Affiliations:** ^1^Epidemic Intelligence Service, CDC; ^2^Division of Violence Prevention, National Center for Injury Prevention and Control, CDC; ^3^Office of the Global AIDS Coordinator, U.S. Department of State, Washington, DC; ^4^CDC Uganda; ^5^CDC Nigeria; ^6^CDC Malawi; ^7^CDC Zambia; ^8^CDC Lesotho; ^9^Federal Ministry of Women Affairs and Social Development, Nigeria; ^10^School of Humanities and Social Sciences, University of Zambia; ^11^Central Statistical Office, Zambia; ^12^Ministry of Gender, Labour and Social Development, Uganda.

Violence is a major public health and human rights concern, claiming over 1.3 million lives globally each year (*1*). Despite the scope of this problem, population-based data on physical and sexual violence perpetration are scarce, particularly in low-income and middle-income countries (*2*,*3*). To better understand factors driving both children becoming victims of physical or sexual violence and subsequently (as adults) becoming perpetrators, CDC collaborated with four countries in sub-Saharan Africa (Malawi, Nigeria, Uganda, and Zambia) to conduct national household surveys of persons aged 13–24 years to measure experiences of violence victimization in childhood and subsequent perpetration of physical or sexual violence. Perpetration of physical or sexual violence was prevalent among both males and females, ranging among males from 29.5% in Nigeria to 51.5% in Malawi and among females from 15.3% in Zambia to 28.4% in Uganda. Experiencing physical, sexual, or emotional violence in childhood was the strongest predictor for perpetrating violence; a graded dose-response relationship emerged between the number of types of childhood violence experienced (i.e., physical, sexual, and emotional) and perpetration of violence. Efforts to prevent violence victimization need to begin early, requiring investment in the prevention of childhood violence and interventions to mitigate the negative effects of violence experienced by children.

From 2013 to 2015, CDC collaborated with Together for Girls* and the governments of Malawi, Nigeria, Uganda, and Zambia to plan and implement Violence Against Children Surveys, which are nationally representative, multistage cluster surveys of adolescents and young adults aged 13–24 years. Surveys were administered via household, face-to-face interviews by host country interviewers trained by CDC and host country partners. Informed consent or assent was obtained for all participants. Multiple safeguards were incorporated into study protocols to protect the confidentiality and safety of participants, including provision of a list of available services for all participants and direct referral to social services for any victims requesting aid.^†^ Study protocols were approved by host country and CDC institutional review boards.

This analysis examines lifetime perpetration of physical or sexual violence among persons of both sexes aged 13–24 years. Physical violence perpetration included ever punching, kicking, whipping, beating, choking, smothering, threatening with a weapon, attempting to drown, or intentionally burning another person. Sexual violence perpetration included forcing nonconsensual sexual intercourse or any other sex acts on another person. In Nigeria, Uganda, and Zambia, sex was defined as vaginal/anal penetration by the penis, hands, fingers, mouth, or objects, or oral penetration by the penis. In Malawi, sex was defined as vaginal, oral, or anal sex or the insertion of an object into an anus or vagina. Prevalence of physical and sexual violence was stratified by perpetration against an intimate partner versus a nonpartner.

Childhood experiences of violence victimization were also examined. Physical violence victimization was defined as ever being punched, kicked, whipped, beaten, choked, smothered, threatened with a weapon, held under water (attempted drowning), or intentionally burned by any person before age 18 years. Emotional violence victimization was defined as ever being told by one’s parents or caregivers that he or she was not loved, that they wished he or she had never been born, or he or she was ridiculed or belittled before age 18 years. Sexual violence victimization was defined as unwanted sexual touching, unwanted attempted sex, physically forced sex, or pressured sex by any person.

Questionnaires for all countries included identical questions regarding perpetration of violence, demographics, and potential risk factors, such as experiences of violence in childhood and educational status. Questionnaires were administered in local languages appropriate to each of the four countries (Malawi: Chichewa and Tumbuka; Nigeria: English, Hausa, Igbo, and Yoruba; Uganda: English, Ateso-Karamajong, Luganda, Lugbara, Luo, Swahili, Runyankole-Rukiga, and Runyoro-Rutoro; and Zambia: English, Bemba, Kaonde, Lozi, Lunda, Luvale, Nyanja, and Tonga). The English survey instrument was translated into local languages, back-translated into English, and cross-validated by a language translation team prior to administration. In addition, the questionnaire was piloted in each country to ensure that the intent of questions was consistent after translation. Weighted percentages of participants reporting lifetime perpetration of physical or sexual violence were calculated for each independent variable. Logistic regression models were used to identify predictors of violence perpetration, adjusting for age at time of survey, marital status, sex, educational status, and experiencing any violence in childhood. To identify independent predictors of perpetration, adjusted models included all significant (p<0.05) factors in unadjusted analyses. Each type of childhood violence was entered in the model separately because of significant collinearity among types of violence. Analyses and data visualizations were conducted using SAS (version 9.4; SAS Institute).

Prevalence of violence perpetration varied by type of violence and country (Table 1). Perpetration of physical violence was more common than sexual violence in all four countries and occurred among both males and females. Perpetration of physical violence was more prevalent among youths in Uganda; 46.2% of males and 26.8% of females in Uganda reported ever perpetrating physical violence against another person. Physical violence against a nonpartner was more common than against an intimate partner in all countries. In contrast, sexual violence was more commonly perpetrated against an intimate partner. Perpetration of sexual violence was most prevalent among males in Malawi; more than one in four (26.6%) males in Malawi reported perpetrating forced sex.

**TABLE 1:** Prevalence of physical and sexual violence perpetration among persons aged 13–24 years, by sex and type of violence — Malawi, Nigeria, Uganda, and Zambia, 2013–2015

Country (survey year)/Violence type	Weighted % (95% CI)	
	Males	Females
**Malawi (2013) N = 1,553**		
Any physical violence	38.6 (33.3–43.8)	23.1 (16.0–30.2)
Physical violence against an intimate partner	8.0 (5.7–10.3)	7.6 (3.2–12.0)
Physical violence against other	34.1 (29.0–39.1)	17.7 (13.3–22.1)
Any forced sex	26.6 (21.9–31.3)	5.9 (1.8–10.1)
Forced sex against an intimate partner	24.5 (19.9–29.2)	4.6 (2.1–7.1)
Forced sex against other	7.5 (5.4–9.7)	2.8 (0.0–7.0)
Any physical or sexual violence	51.5 (46.1–57.0)	25.6 (18.7–32.6)
**Nigeria (2014) N = 2,464**		
Any physical violence	24.8 (21.3–28.4)	19.0 (15.1–22.8)
Physical violence against an intimate partner	13.5 (10.8–16.0)	6.3 (4.5–8.2)
Physical violence against other	18.1 (15.0–21.2)	13.8 (10.5–17.0)
Any forced sex	8.5 (6.1–10.9)	1.5 (0.8–2.3)
Forced sex against an intimate partner	6.4 (4.4–8.5)	1.2 (0.5–1.9)
Forced sex against other	3.3 (1.8–4.8)	0.7 (0.2–1.2)
Any physical or sexual violence	29.5 (25.5–33.5)	19.9 (16.1–23.7)
**Uganda (2015) N = 3,875**		
Any physical violence	46.2 (43.3–49.1)	26.8 (22.5–31.0)
Physical violence against an intimate partner	18.7 (16.3–21.1)	7.6 (5.1–10.0)
Physical violence against other	37.7 (35.0–40.5)	22.9 (19.0–26.9)
Any forced sex	11.7 (9.8–13.6)	2.1 (1.1–3.1)
Forced sex against an intimate partner	9.5 (7.6–11.3)	2.1 (1.1–3.1)
Forced sex against other	4.7 (3.6–5.8)	0.1 (0.0–0.3)
Any physical or sexual violence	50.6 (47.8–53.5)	28.4 (24.1–32.6)
**Zambia (2014) N = 1,170**		
Any physical violence	23.7 (19.5–28.0)	12.5 (9.3–15.7)
Physical violence against an intimate partner	13.9 (10.4–17.4)	7.4 (5.0–9.9)
Physical violence against other	17.9 (14.1–21.7)	7.5 (5.0–10.0)
Any forced sex	14.6 (10.5–18.7)	3.5 (1.6–5.5)
Forced sex against an intimate partner	12.0 (8.3–15.8)	3.1 (1.2–5.0)
Forced sex against other	6.7 (4.2–9.2)	1.7 (0.1–3.2)
Any physical or sexual violence	32.8 (28.1–37.6)	15.3 (12.1–18.6)

**Abbreviation:** CI = confidence interval.

Among respondents in all four countries, males and victims of childhood violence had consistently higher odds of perpetrating physical or sexual violence (Table 2). In all countries, being a victim of childhood violence was the strongest independent predictor of being a perpetrator of violence. In all countries, the adjusted odds ratio (aOR) for perpetrating violence was more than five times higher (aOR range = 5.4–7.0) for victims of childhood violence, compared with those who had not experienced violence in childhood. Experiencing physical violence in childhood was associated with the highest odds of perpetrating any form of violence in all countries (aOR range = 2.8–6.4). Experiencing childhood sexual or physical violence was consistently associated with similar types of violence perpetration across all countries when stratified by sex.

**TABLE 2:** Prevalence and adjusted odds ratios for physical and sexual violence perpetration among persons aged 13–24 years, by risk factors for perpetrating violence — Malawi, Nigeria, Uganda, and Zambia, 2013–2015

Country (survey year)/Risk factor	Ever perpetrated physical or sexual violence, weighted % (95% CI)	Chi-square p-value	OR (95% CI)	Adjusted OR (95% CI)
Malawi* (2013) N = 1,553				
**Age at time of survey (yrs)**				
13–17	51.4 (43.5–59.4)	<0.001	2.3 (1.6–3.3)	1.4 (0.9–2.3)
18–24	31.3 (25.8–36.9)		1.0 (Ref)	1.0 (Ref)
**Marital status**				
Ever married or living as married	25.8 (20.4–31.1)	<0.001	0.4 (0.3–0.5)	0.8 (0.5–1.2)
Never married	47.6 (41.9–53.3)		1.0 (Ref)	1.0 (Ref)
**Education**				
Primary education or less	36.6 (30.5–42.7)	0.38	0.8 (0.6–1.2)	—
Secondary or more	40.6 (33.4–47.8)		1.0 (Ref)	
**Sex**				
Male	51.5 (46.1–57.0)	<0.001	3.1 (2.0–4.7)	2.6 (1.6–4.3)
Female	25.6 (18.7–32.6)		1.0 (Ref)	1.0 (Ref)
**Victim of any violence in childhood^†^**				
Yes	45.5 (39.9–51.1)	<0.001	8.4 (5.1–13.8)	7.0 (4.1–12.0)
No	9.1 (5.1–13.0)		1.0 (Ref)	1.0 (Ref)
**Victim of sexual violence in childhood**				
Yes	47.8 (37.2–58.4)	0.01	1.9 (1.2–3.1)	2.6 (1.5–4.4)^§^
No	32.6 (27.2–38.0)		1.0 (Ref)	1.0 (Ref)
**Victim of physical violence in childhood**				
Yes	46.0 (41.0–51.0)	<0.001	3.8 (2.4–6.1)	2.8 (1.7–4.5)
No	18.2 (11.0–25.5)		1.0 (Ref)	1.0 (Ref)
**Victim of emotional violence in childhood**				
Yes	55.7 (50.0–61.4)	<0.001	2.9 (2.1–4.0)	2.5 (1.8–3.4)
No	30.0 (24.6–35.4)		1.0 (Ref)	1.0 (Ref)
**Nigeria^¶^ (2014) N = 2,464**				
**Age at time of survey (yrs)**				
13–17	32.8 (27.8–37.9)	<0.001	1.9 (1.4–2.4)	1.7 (1.2–2.2)
18–24	20.8 (17.8–23.7)		1.0 (Ref)	1.0 (Ref)
**Marital status**				
Ever married or living as married	15.5 (12.3–18.7)	<0.001	0.4 (0.3–0.6)	0.6 (0.5–0.9)
Never married	29.5 (26.0–33.1)		1.0 (Ref)	1.0 (Ref)
**Education**				
Primary education or less	19.2 (14.6–23.7)	0.01	0.7 (0.5–0.9)	1.0 (0.7–1.4)
Secondary or more	26.6 (23.2–30.0)		1.0 (Ref)	1.0 (Ref)
**Sex**				
Male	29.5 (25.5–33.5)	<0.001	1.7 (1.2–2.3)	1.5 (1.1–2.1)
Female	19.9 (16.1–23.7)		1.0 (Ref)	1.0 (Ref)
**Victim of any violence in childhood**				
Yes	31.4 (27.8–35.0)	<0.001	7.3 (4.9–10.8)	6.6 (4.4–9.8)
No	5.9 (3.9–7.9)		1.0 (Ref)	1.0 (Ref)
**Victim of sexual violence in childhood**				
Yes	31.2 (26.2–36.2)	<0.001	1.6 (1.3–2.2)	1.7 (1.3–2.2)
No	21.6 (18.5–24.6)		1.0 (Ref)	1.0 (Ref)
**Victim of physical violence in childhood**				
Yes	34.7 (30.7–38.7)	<0.001	5.7 (4.2–7.8)	5.2 (3.8–7.1)
No	8.5 (6.4–10.6)		1.0 (Ref)	1.0 (Ref)
**Victim of emotional violence in childhood**				
Yes	35.7 (30.6–40.7)	<0.001	2.1 (1.7–2.8)	1.9 (1.4–2.5)
No	20.6 (17.5–23.6)		1.0 (Ref)	1.0 (Ref)
Uganda** (2015) N = 3,875				
**Age at time of survey (yrs)**				
13–17	57.6 (53.3–62.0)	<0.001	3.1 (2.5–3.8)	2.2 (1.7–2.8)
18–24	30.8 (27.5–34.1)		1.0 (Ref)	1.0 (Ref)
**Marital status**				
Ever married or living as married	28.9 (25.3–32.4)	<0.001	0.4 (0.4–0.6)	0.9 (0.7–1.2)
Never married	47.7 (43.7–51.7)		1.0 (Ref)	1.0 (Ref)
**Education**				
Primary education or less	36.7 (31.5–41.8)	0.35	1.1 (0.9–1.4)	—
Secondary or more	39.4 (36.1–42.6)		1.0 (Ref)	
**Sex**				
Male	50.6 (47.8–53.5)	<0.001	2.6 (2.0–3.3)	2.5 (1.9–3.2)
Female	28.4 (24.1–32.6)		1.0 (Ref)	1.0 (Ref)
**Victim of any violence in childhood**				
Yes	43.8 (40.6–47.1)	<0.001	7.1 (5.1–9.9)	6.5 (4.7–8.9)
No	10.3 (7.2–13.5)		1.0 (Ref)	1.0 (Ref)
**Victim of sexual violence in childhood**				
Yes	40.6 (35.0–46.1)	0.31	1.1 (0.9–1.5)	1.3 (1.0–1.7)
No	37.3 (33.9–40.7)		1.0 (Ref)	1.0 (Ref)
**Victim of physical violence in childhood**				
Yes	48.7 (45.2–52.2)	<0.001	7.6 (5.7–10.2)	6.4 (4.8–8.5)
No	11.1 (8.4–13.9)		1.0 (Ref)	1.0 (Ref)
**Victim of emotional violence in childhood**				
Yes	47.7 (43.2–52.3)	<0.001	2.0 (1.6–2.4)	1.9 (1.5–2.3)
No	31.7 (28.4–35.0)		1.0 (Ref)	1.0 (Ref)
**Zambia^††^ (2014) N = 1,170**				
**Age at time of survey (yrs)**				
13–17	27.2 (21.1–33.2)	0.07	1.4 (1.0–2.0)	1.1 (0.7–1.8)
18–24	21.1 (17.8–24.4)		1.0 (Ref)	1.0 (Ref)
**Marital status**				
Ever married or living as married	17.3 (13.4–21.1)	0.001	0.6 (0.4–0.8)	0.9 (0.6–1.4)
Never married	26.8 (22.5–31.0)		1.0 (Ref)	1.0 (Ref)
**Education**				
Primary education or less	19.5 (15.3–23.7)	0.05	0.7 (0.5–1.0)	0.8 (0.5–1.1)
Secondary or more	25.6 (21.3–29.9)		1.0 (Ref)	1.0 (Ref)
**Sex**				
Male	32.8 (28.1–37.6)	<0.001	2.7 (1.9–3.8)	2.5 (1.8–3.6)
Female	15.3 (12.1–18.6)		1.0 (Ref)	1.0 (Ref)
**Victim of any violence in childhood**				
Yes	31.6 (27.5–35.7)	<0.001	5.4 (3.4–8.6)	5.4 (3.4–8.6)
No	7.9 (4.9–10.9)		1.0 (Ref)	1.0 (Ref)
**Victim of sexual violence in childhood**				
Yes	32.5 (26.0–39.0)	<0.001	1.9 (1.3–2.8)	2.6 (1.8–3.9)^§§^
No	19.8 (16.4–23.3)		1.0 (Ref)	1.0 (Ref)
**Victim of physical violence in childhood**				
Yes	37.0 (31.8–42.3)	<0.001	5.1 (3.5–7.5)	4.9 (3.3–7.2)
No	10.3 (7.5–13.2)		1.0 (Ref)	1.0 (Ref)
**Victim of emotional violence in childhood**				
Yes	42.4 (34.5–50.2)	<0.001	3.6 (2.4–5.4)	3.4 (2.3–5.1)
No	16.8 (13.8–19.7)		1.0 (Ref)	1.0 (Ref)

**Abbreviations:** CI = confidence interval; OR = odds ratio; Ref = referent.

* Malawi model included age at time of survey, marital status, and sex. Effect measure modification between sex, age, and sexual violence was observed for Malawi; pooled results are provided.

^†^ For all four countries, violence victimization was defined as ever experiencing any physical, sexual, or emotional violence at age <18 years.

^§^ Stratified adjusted ORs: females: 2.3 (0.9–5.9), males: 3.1 (1.8–5.2); age 13–17 years: 1.4 (0.8–2.5), age 18–24 years: 3.9 (2.0–7.6).

^¶^ Nigeria model included age at time of survey, marital status, educational status, and sex.

** Uganda model included age at time of survey, marital status, and sex.

^††^ Zambia model included age at time of survey, marital status, educational status, and sex. Effect measure modification between sex and sexual violence was observed for Zambia; pooled results are provided.

^§§^ Stratified adjusted ORs: females, 2.2 (1.3–3.8); males, 3.1 (1.8–5.5).

A dose-response relationship between the number of types of violence experienced in childhood and adjusted odds of perpetrating violence was observed for all countries (Figure). For example, in Zambia, persons who experienced physical, emotional, and sexual violence before age 18 years were approximately 20 times more likely to perpetrate violence than were persons who did not experience any form of violence (aOR = 19.8, 95% confidence interval = 9.0–43.6).

**FIGURE:**
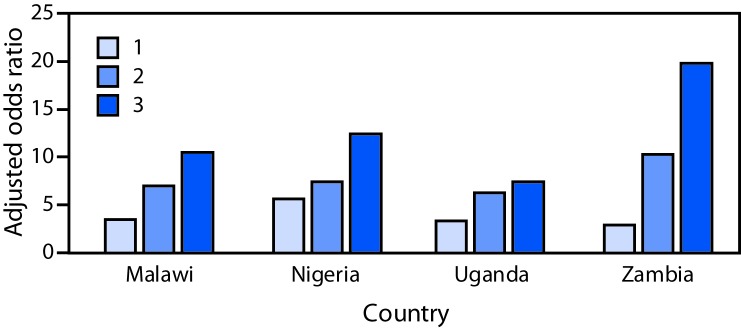
Adjusted odds ratios for perpetrating violence based on the number of types of violence* experienced in childhood,^†^ among persons aged 13–24 years — four sub-Saharan African countries,^§^ 2013–2015 * The three types of violence analyzed were physical, sexual, and emotional violence. The referent was experiencing zero types of violence. ^†^ Experienced at age <18 years. ^§^ The Malawi model included age at time of survey, marital status, sex, and victim of violence in childhood status; the Nigeria model included age at time of survey, marital status, educational status, sex, and victim of violence in childhood status; the Uganda model included age at time of survey, marital status, sex, and victim of violence in childhood status; the Zambia model included age at time of survey, marital status, educational status, sex, and victim of violence in childhood status.

## Discussion

Perpetration of violence is common among both males and females in Malawi, Nigeria, Uganda, and Zambia. Strong associations between youths’ experiences of violence and subsequent perpetration of physical or sexual violence were observed in all four studied countries. The dose-response relationship observed between the number of types of violence experienced in childhood and the odds of perpetrating violence highlights the importance of interrupting the cycle of violence early in life.

Previous population-based studies of men in Brazil, Chile, Croatia, India, Mexico, Rwanda, and South Africa estimated lifetime prevalence of physical intimate partner violence perpetration at 24%–42% (*4*,*5*). Accurately quantifying and addressing perpetration of violence is a critical first step to reducing such violence, along with its associated consequences, such as transmission of human immunodeficiency virus (*6*,*7*).

Associations between experiences of physical, sexual, and emotional violence during childhood and subsequent perpetration of violence are consistent with a growing body of research linking adverse childhood experiences with later perpetration of criminal violence, child abuse, and intimate partner violence (*8*,*9*). Understanding the risk factors for perpetration is integral to combating violence, particularly in stopping the transmission cycle of violence. Efforts to prevent future violence must include both strategies to prevent perpetration and interventions to counteract the negative effects of physical, sexual, and emotional violence among victims. Improved data and information on what factors buffer victims of violence from potential adverse consequences can inform the development and evaluation of programs and policies to interrupt the intergenerational cycle of violence.

The findings in this report are subject to at least five limitations. First, this was a cross-sectional study, and causality between violence victimization and perpetration cannot be established. Second, data were self-reported, and recall bias might be present, particularly for remote episodes of abuse. Third, limited disclosure might have occurred because of the sensitive nature of the survey, particularly questions asking about perpetration. Fourth, surveys did not assess the frequency of violent acts committed by perpetrators or the interval between victimization and perpetration; future studies of the links between victimization and perpetration could benefit from inclusion of this information. Finally, persons not living in households (e.g., street children, children living in institutions, or students living in dormitories) were not included in this analysis; therefore, these findings might not be generalizable.

The strong association between experiencing physical, sexual, or emotional violence in childhood and later perpetration of violence highlights the importance of long-term, comprehensive interventions for both victims and perpetrators. Potential strategies include improved access to therapeutic services and counseling, support and education of parents, reduction of community violence, and improving gender equity (*10*). In addition, the unique results observed for different countries emphasize the need for country-specific data to respond directly to countries’ distinct patterns and drivers of violence. Although violence perpetration is common among both males and females, it is preventable. INSPIRE is a technical package that aids countries in identifying evidence-based programs and policies to prevent and respond to violence against children (*10*). Early intervention is critical to preventing the adverse effects of violence victimization.

**Summary**

**What is already known about this topic?**

Violence against children is a public health issue with important consequences, including the subsequent potential perpetration of violence by victims.

**What is added by this report?**

Analysis of data from Violence Against Children Surveys in four sub-Saharan African countries found that the prevalence of violence perpetration ranged among males from 29.5% in Nigeria to 51.5% in Malawi and among females from 15.3% in Zambia to 28.4% in Uganda. In all countries, a strong dose-response relationship was observed between the number of types of childhood violence experienced and odds of perpetrating violence.

**What are the implications for public health practice?**

The strong association between experiencing violence in childhood and later perpetration of violence highlights the importance of long-term, comprehensive interventions for both victims and perpetrators.

## Footnotes

* Together for Girls is a public-private partnership comprising host country governments, the United Nations Children’s Fund, the President’s Emergency Plan for AIDS Relief, and other organizations. https://www.togetherforgirls.org.

†
https://www.cdc.gov/violenceprevention/pdf/vacs/VACS-trainingwhitepaper.pdf .
